# The Zoonotic Helminth Parasite *Fasciola hepatica:* Virulence-Associated Cathepsin B and Cathepsin L Cysteine Peptidases Secreted by Infective Newly Excysted Juveniles (NEJ)

**DOI:** 10.3390/ani11123495

**Published:** 2021-12-08

**Authors:** Tara Barbour, Krystyna Cwiklinski, Richard Lalor, John Pius Dalton, Carolina De Marco Verissimo

**Affiliations:** 1 School of Biological Science, Queen’s University Belfast, Belfast BT9 7BL, UK; t.barbour@qub.ac.uk (T.B.); krystyna.cwiklinski@nuigalway.ie (K.C.); johnpius.dalton@nuigalway.ie (J.P.D.); 2 Molecular Parasitology Laboratory, Centre for One Health and Ryan Institute, School of Natural Sciences, National University of Ireland Galway, H91 TK33 Galway, Ireland; richard.lalor@nuigalway.ie

**Keywords:** *Fasciola hepatica*, liver fluke, trematode, flatworm, parasites, cysteine peptidases, cathepsin L, cathepsin B, drug targets, vaccines

## Abstract

**Simple Summary:**

Fasciolosis, caused by the worm parasite *Fasciola hepatica* (liver fluke), is a global disease of farm animals and a neglected disease of humans. Infection arises from the ingestion of resistant metacercariae that contaminate vegetation. Within the intestine, the parasite excysts as an active larvae, the newly excysted juvenile (NEJ), that borrows through the intestinal wall to infect the host and migrates to the liver. NEJ release, tissue penetration and migration are facilitated by enzymes secreted by the parasite, namely, cathepsin B1 (FhCB1), cathepsin B2 (FhCB2), cathepsin B3 (FhCB3) and cathepsin L3 (FhCL3). While our knowledge of these enzymes is growing, we have yet to understand why the parasites require all four of them to invade the host. In this study, we produced functional recombinant forms of these enzymes and demonstrated that they vary greatly in terms of activity, optimal pH and substrate specificity, suggesting that, combined, these enzymes provide the parasite with an efficient digestion system for different host tissues and molecules. We also identified several compounds that inhibited the activity of these enzymes, but did not affect the ability of the larvae to excyst or survive. However, this does not exclude these enzymes as targets for development of drugs or vaccines.

**Abstract:**

Fasciolosis caused by *Fasciola hepatica* is a major global disease of livestock and an important neglected helminthiasis of humans. Infection arises when encysted metacercariae are ingested by the mammalian host. Within the intestine, the parasite excysts as a newly excysted juvenile (NEJ) that penetrates the intestinal wall and migrates to the liver. NEJ excystment and tissue penetration are facilitated by the secretion of cysteine peptidases, namely, cathepsin B1 (FhCB1), cathepsin B2 (FhCB2), cathepsin B3 (FhCB3) and cathepsin L3 (FhCL3). While our knowledge of these peptidases is growing, we have yet to understand why multiple enzymes are required for parasite invasion. Here, we produced functional recombinant forms of these four peptidases and compared their physio-biochemical characteristics. Our studies show great variation of their pH optima for activity, substrate specificity and inhibitory profile. Carboxy-dipeptidase activity was exhibited exclusively by FhCB1. Our studies suggest that, combined, these peptidases create a powerful hydrolytic cocktail capable of digesting the various host tissues, cells and macromolecules. Although we found several inhibitors of these enzymes, they did not show potent inhibition of metacercarial excystment or NEJ viability in vitro. However, this does not exclude these peptidases as targets for future drug or vaccine development.

## 1. Introduction

Fasciolosis caused by the parasite *Fasciola hepatica*, or liver fluke, is a food- and water-borne disease of humans and their livestock. The parasite has a global distribution and results in major losses to the agricultural community, conservatively placed at EUR 2.5 billion each year, due to the reduced productivity of infected sheep, cattle, water buffalo and goats [1,2]. Infections of humans are estimated to be between 2.4 and 17 million, while >180 million people across 70 countries live at risk of infection [3]. Recently, the disability-adjusted life years (DALYs) for this disease has been estimated at 35,000 per annum [3,4] and it is now recognised as a major neglected helminth infection by the World Health Organisation [3].

The parasitic flatworm infects its mammalian host following ingestion of the metacercariae life stage, which is found encysted on vegetation, such as grass or edible aquatic plants and floating in water. A newly excysted juvenile (NEJ) emerges from the cyst in the small intestine and quickly penetrates the wall of the intestinal epithelium and surrounding tissues to make its way to the liver [5]. Here, the parasite spends about two months tunnelling and feeding on the liver parenchymal tissue before moving into the bile ducts, where it establishes a chronic infection, becomes fecund and produces thousands of eggs each day. These eggs are carried with bile secretions to the intestine and then passed within the faeces into the external surrounding environment where they fully develop and hatch to release a miracidium that is infectious to aquatic snails such as *Galba truncatula*. After a series of asexual cloning and developmental steps within the snail, the parasites emerge as cercariae and then settle as encysted metacercariae to complete the cycle [6,7,8].

Parasites that are resistant to frontline anti-liver fluke treatments, such as triclabendazole, have spread globally, causing concern for the control of the disease, particularly since there are no commercially available vaccines. For the development of new control treatments, detailed molecular biological studies are required to identify amenable targets in the parasite. Recently, much research has focused on the NEJ stage and its means of invasion, since it is this stage that initiates infection and leads to the more damaging later developmental stages. Proteomic and transcriptomic studies have revealed the identity of several peptidases that the NEJ secrete to facilitate infection by breaking down the molecules, cells and tissues of the intestinal wall [5,9,10,11]. These peptidases include four papain-like cysteine peptides, three *F. hepatica* cathepsins B (FhCBs), termed FhCB1, FhCB2 and FhCB3 [9,12,13,14,15], and a cathepsin L, termed FhCL3 [9,16,17].

Structural studies of papain-like cysteine peptidases show that they share a common fold [18]. Mature enzymes are bi-lobed molecules consisting of two domains with an active site located in the cleft between them. Substrates bind to the peptidases via a series of active site subsites (S1, S2, S3, etc.) that determine the specificity of the enzyme. The proteolytic activity of cysteine peptidases is conferred by the presence of three active site residues, Cys-His-Asn, known as the catalytic triad, which are part of the S1 subsite [19]. The carbonyl group of the substrate P1 residue is located in an oxyanion hole next to the active site cysteine whose thiol group acts as a nucleophile in enzyme–substrate interactions, while the P1 side chain is orientated outwards toward the solvent. The backbone amides of the substrate residues at positions P2, P1 and P1′ form a network of hydrogen bonds with surface residues in the corresponding substrate subsites S2, S1 and S1′ [18,20,21,22,23,24].

The cathepsins B and L are produced as zymogens that possess an N-terminal propeptide region that acts as an inhibitor of their cognate peptidase by acting as a ‘clamp’ to block the peptidase active site. Removal of the propeptide may occur either auto- or trans-catalytically, at acidic pH [25,26,27]. We have shown that the cathepsins B and L of *F. hepatica* NEJs are located in the gastrodermal cells lining the parasite gut from which they are secreted into the low pH environment of the gut lumen [9,28,29].

To date, no comparative study of the secreted cathepsins B and cathepsins L from the infective *F. hepatica* NEJs has been published. Therefore, in the present study, we conducted a physio-biochemical characterisation of the endopeptidase activity of the recombinantly produced FhCB1, FhCB2, FhCB3 and FhCL3 to probe their specificity and identify differences and novelties that might help elucidate the roles of these enzymes in the invasiveness and pathogenicity of *F. hepatica*.

## 2. Material and Methods

### 2.1. Production, Purification and In Vitro Autocatalytic Processing of Recombinant FhCBs and FhCL3

The recombinant proteins, FhCB1, FhCB2, FhCB3 and FhCL3, were produced in the methylotrophic yeast *Pichia pastoris* and isolated from the medium by Nickle-chelate affinity chromatography as described previously [27]. Autocatalytic activation of recombinant proenzymes to their mature forms was carried out in C-P activation buffer (0.1 M citrate-phosphate, 100 mM NaCl and 2 mM DTT, at pH 4.5) supplemented with dextran sulphate (10 μg/mL) at a concentration of 100 μg/mL at 37 °C for 2 h. Activated proteins were dialysed against phosphate-buffered saline (PBS; Sigma-Aldrich, St. Louis, MO, USA) and stored at −20 °C.

The *Schistosoma mansoni* cathepsin B1 (SmCB1) was also produced in our laboratory using *P. pastoris* as described above. The human cathepsin B (HsCB) and cathepsin L1 (HsCL1) were produced in human embryonic kidney cells (Sigma-Aldrich).

### 2.2. Assessment of Enzymatic Activity by Fluorogenic Substrate Assay

Recombinant peptidases were assayed for enzymatic activity using a fluorogenic substrate assay. The reactions were carried out in 0.1 M C-P buffer (pH 7.0) with 1 mM DTT in a final volume of 200 μL, in a black 96-well plate. Each well contained activated enzyme (140 nM) and 20 μM fluorogenic substrate, which was added to start the reaction. All assays were run in triplicate at 37 °C for 1 h. Enzyme activity was monitored by rate of hydrolysis and subsequent release of the AMC group on the synthetic peptide substrates at an excitation wavelength of 370 nm and an emission wavelength of 460 nm, as relative fluorescence units (RFU), in a PolarStar Omega Spectrophotometer (BMG Labtech, Aylesbury, UK). All data were plotted and analysed using MARS Data Analysis Software (BMG Labtech, UK) and GraphPad Prism version 5.

To determine pH optima for activity, fluorescence assays were performed in 0.1 M C-P buffer with 1 mM DTT at a range of pH of 3.5–8.0. The fluorogenic substrates used were Z-Phe-Arg-AMC (for FhCB1 and FhCB3) and Z-Gly-Pro-Arg-AMC (for FhCB2 and FhCL3).

The substrate specificities of the recombinant *F. hepatica* peptidases were investigated using a panel of six fluorogenic peptide substrates (Bachem, St Helens, UK), Z-Phe-Arg-AMC, Z-Arg-Arg-AMC, Z-Leu-Arg-AMC, Z-Gly-Pro-Arg-AMC, Z-Pro-Arg-AMC and Z-Val-Ile-Arg-AMC in 0.1 M C-P buffer with 1 mM DTT, at the optimum pH for each peptidase.

To determine the kinetic constants, *K*_cat_, *K*_M_ and *K*_cat_/*K*_M_ of the activated peptidases against the Z-Phe-Arg-AMC, Z-Gly-Pro-Arg-AMC and Z-Val-Ile-Arg-AMC substrates were used at concentrations of 2000–4 μM over a series of 10 dilutions using enzymes, buffer and assay conditions as described above. The kinetic constants, *K*_M_ and *V*_max_, were calculated using the Michaelis–Menten equation (Equation (1)) in a non-linear regression analysis of initial reaction velocity against substrate concentration (built into GraphPad Prism 5). The value of *K*_M_ can then be fitted to Equation (2) to determine the catalytic constant *K*_cat_.

(1) $$\nu =\frac{d\left[P\right]}{dt}=\frac{V_{\max}\left[S\right]}{K_{M}+\left[S\right]}$$

where *ν* is the initial reaction velocity, [*P*] is the concentration of the formed product, [S] is the substrate concentration, *V*_max_ is the maximum rate achieved by the system and *K*_M_ is the Michaelis–Menten constant, which represents the concentration of substrate at which the reaction has a rate equal to half of *V*_max_.

(2) $$\nu =\frac{K_{\mathrm{cat}}\left[E_{t}\right]\{\}\left[S\right]}{K_{M}+\left[S\right]}$$

where [E*_t_*] is the enzyme concentration and *K*_cat_ is the catalytic constant, representing the measure of substrate molecules converted to product by enzyme per second.

### 2.3. Determination of Inhibitor Specificity against Recombinant FhCBs and FhCL3

Commercially available inhibitors of cathepsin B (Ca074 and Ca074-OMe), cathepsin L (L, L1, LII, LIII and LIV), cathepsin K (KI, KII and KIII) and cathepsin S were purchased from Merck Millipore (Cork, Ireland; Supplementary Figure S2). The broad-spectrum papain peptidase inhibitor Z-Phe-Ala-FMK was purchased from Enzo Life Sciences (Exeter, UK). These were used to determine and compare the inhibition constant (*K*_i_) against the recombinant FhCBs and FhCL3. All the assays were carried out in 0.1 M C-P buffer with 1 mM DTT and 0.01% Brij^®^ L23, at the pH corresponding to the optimum for each enzyme in a total 200 µL reaction volume. The peptidases (140 nM) were pre-incubated with the inhibitor (1000-15 nM) at room temperature, for 30 min, before starting the reaction by the addition of the substrate that was optimal for each enzyme (FhCB1, Z-Phe-Arg-AMC; FhCB2 and FhCB3, Z-Val-Ile-Arg-AMC; FhCL3, Z-Gly-Pro-Arg-AMC). Substrate concentrations were equal to *K*_M_ (shown in Table 1) and the apparent inhibition constant (*K*_i_^app^) values were calculated with GraphPad Prism 5, using the Morrison equation for tight binding inhibition (Equation (3)). For competitive inhibitors, such as those used in this study, *K*_i_^app^ was fitted to a second equation (Equation (4)) from which *K*_i_ can be determined [30].

(3) $$\frac{v_{i}}{v_{o}}=1-\frac{\left(\left[E\right]\right)+\left[I\right]+K_{i}^{\mathrm{app}})-\sqrt{{\left(\left[E\right]+\left[I\right]+K_{i}^{\mathrm{app}}\right)}^{2}-4\left[E\right]\left[I\right]\;}}{2\left[E\right]}$$

(4) $$K_{i}^{\mathrm{app}}=K_{i}\left(1+\frac{\left[S\right]}{K_{M}}\right)$$

### 2.4. Exo-Carboxypeptidase Activity of Recombinant FhCBs and FhCL3

The exo-carboxypeptidase activity of the FhCBs and FhCL3 was compared with the activity of the *Schistosoma mansoni* cathepsin B1 (SmCB1), human cathepsin B (HsCB) and cathepsin L (HsCL1) (positive controls). The exopeptidase activity was examined using two fluorescent substrates, Abz-Phe-Arg-Ala-Lys(Dnp)-OH and Abz-Phe-Arg-Ala-Lys(Dnp)-NH_2_, where Abz is 2-aminobenzoyl and Dnp is 2,4-dinitrophenyl (Biomatik, Cambridge, Canada). The peptidases were assayed using a similar protocol to that described above for measuring endopeptidase activity. The reactions were carried out in 0.1 M C-P buffer, pH 5.0, with a final substrate concentration of 20 μM. The enzymatic activity of the peptidases was monitored as RFU, in a PolarStar Omega Spectrophotometer, with readings taken at excitation and emission wavelengths of 355 nm and 460 nm, respectively.

### 2.5. Effects of Inhibitors on the Excystment of F. hepatica Metacercariae and Viability of NEJ

*F. hepatica* metacercariae (Italian isolate; Ridgeway Research, Saint Briavels, UK) were prepared for excystment as previously described by Robinson et al. [10]. To verify the effect of the cathepsin peptidase inhibitors on the ability of the metacercariae to excyst, 50 metacercariae per well were placed into a 24-well plate and 1 mL of excystment medium (0.6% sodium bicarbonate, 0.45% sodium chloride, 0.4% sodium tauroglycocholate, 0.025 N HCl and 0.4% L-cysteine) supplemented with 100 μM of the inhibitor was added to the well. As controls, we used PBS and dimethylsulfoxide (DMSO, 1:100; Sigma-Aldrich) alone. The plate was then incubated at 37 °C with 5% CO_2_, for 3 h to allow metacercarial excystment. Using a light microscope (25× magnification), the number of excysted NEJ was determined.

NEJ were washed three times in a culturing medium (RPMI-1640 medium supplemented with 30 mM HEPES, 0.1% glucose, 10% foetal bovine serum and 50 μg/mL of gentamicin) and their viability assessed by culturing the parasites in 1 mL of culture medium containing 100 μM of the inhibitor at 5% CO_2_ at 37 °C for 24 h. After incubation, the number of moribund/dead parasites were counted using a light microscope (25× magnification). The criteria used to assess the NEJ viability were loss of motility and peristaltic movement and any obvious damage to the tegument or internal structures, as previously standardized by [31]. The experiments were performed in triplicate. The average of the replicate experimental data was subjected to a two-way ANOVA comparing the values obtained with each inhibitor against the mean of the DMSO control group, with minimum 95% confidence intervals. All the statistical analyses were performed using GraphPad Prism 5.

## 3. Results

### 3.1. Production and Isolation of Recombinant F. hepatica FhCB1, FhCB2, FhCB3 and FhCL3

**Figure 1:**
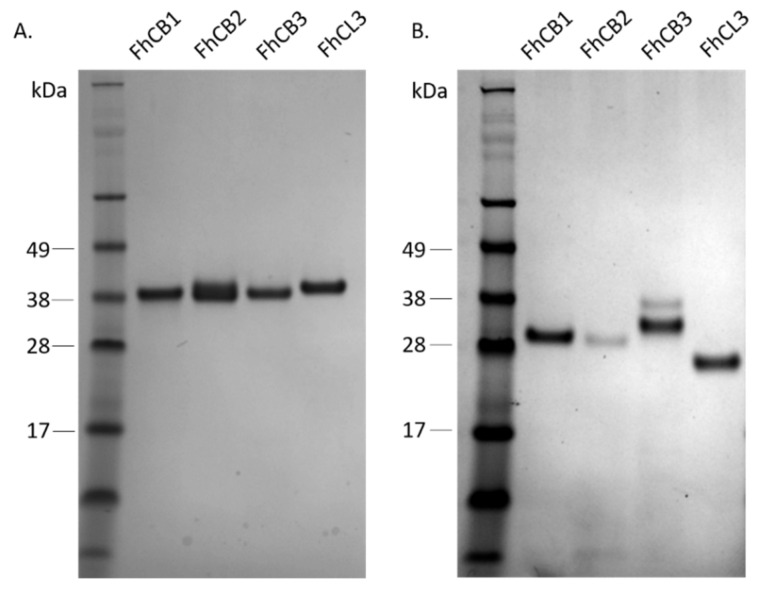
SDS-PAGE analysis of purified recombinant *Fasciola hepatica* newly excysted juvenile (NEJ) cathepsins B (FhCB1, FhCB2, FhCB3) and cathepsin L (FhCL3) peptidases. (**A**) Analysis of purified peptidase zymogens. (**B**) Analysis of mature enzymes following autocatalytic activation.

Utilising the yeast *P. pastoris* system, the recombinant forms of FhCB1, FhCB2, FhCB3 and FhCL3 were produced as zymogens that could be purified to homogeneity using affinity chromatography at a yield from 5 to 15 milligrams per litre. The peptidases were resolved in SDS-PAGE, producing bands in the expected molecular weight of ~37 kDa (Figure 1A). All four proenzymes could be auto-catalytically activated to functional mature forms under acidic conditions, when incubated in C-P activation buffer with the addition of dextran sulphate at pH 4.5 (Figure 1B). The mature cathepsin peptidases were demonstrated to be active enzymes when assayed against fluorogenic peptide substrates (see below).

### 3.2. pH Dependency of Mature F. hepatica FhCB1, FhCB2, FhCB3 and FhCL3

**Figure 2:**
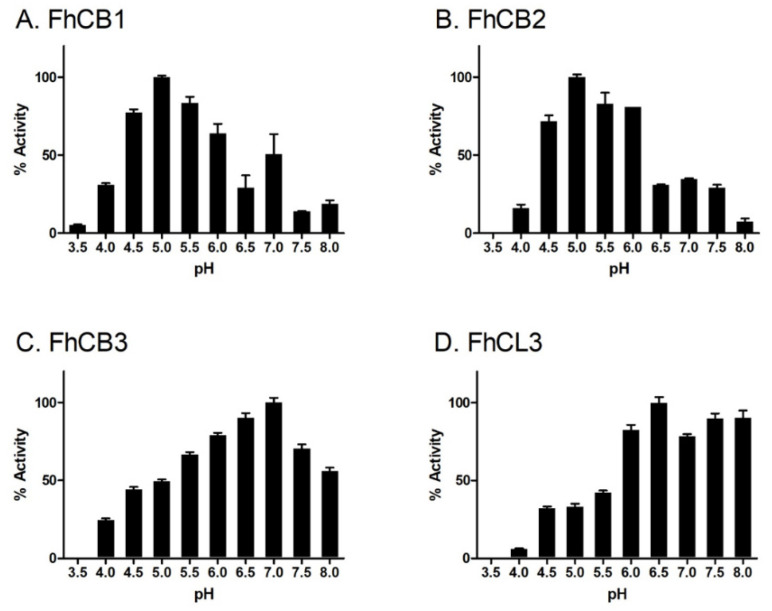
pH optima for activity of FhCB1, FhCB2, FhCB3 and FhCL3. The enzymatic activity of the *F. hepatica* peptidases was assessed in 0.1 M C-P buffer with 1 mM DTT at a pH range 3.5–8.0 using the fluorogenic peptide substrates Z-Phe-Arg-AMC [for FhCB1 (**A**) and FhCB3 (**C**)] and Z-Gly-Pro-Arg-AMC [for FhCB2 (**B**) and FhCL3 (**D**)]. Activity is represented as percentage activity based on the pH where the greatest activity was observed. Error bars indicate standard deviation of three replicates performed in two experiments.

To first establish the pH dependency of FhCB1, FhCB2, FhCB3 and FhCL3, their ability to cleave fluorogenic peptide substrates was determined over the pH range 3.5–8.0 (Figure 2). The various mature peptidases displayed optimum activity at different pH values; FhCB1 and FhCB2 exhibited the greatest enzymatic activity against Z-Phe-Arg-AMC and Z-Gly-Pro-Arg-AMC, respectively, at pH 5.0. By contrast, both FhCB3 and FhCL3 efficiently cleaved Z-Phe-Arg-AMC and Z-Gly-Pro-Arg-AMC at an optimum pH of 7.0 and pH 6.5, respectively.

### 3.3. FhCB1, FhCB2, FhCB3 and FhCL3 Exhibit Distinct Substrate Preference Profiles

**Figure 3:**
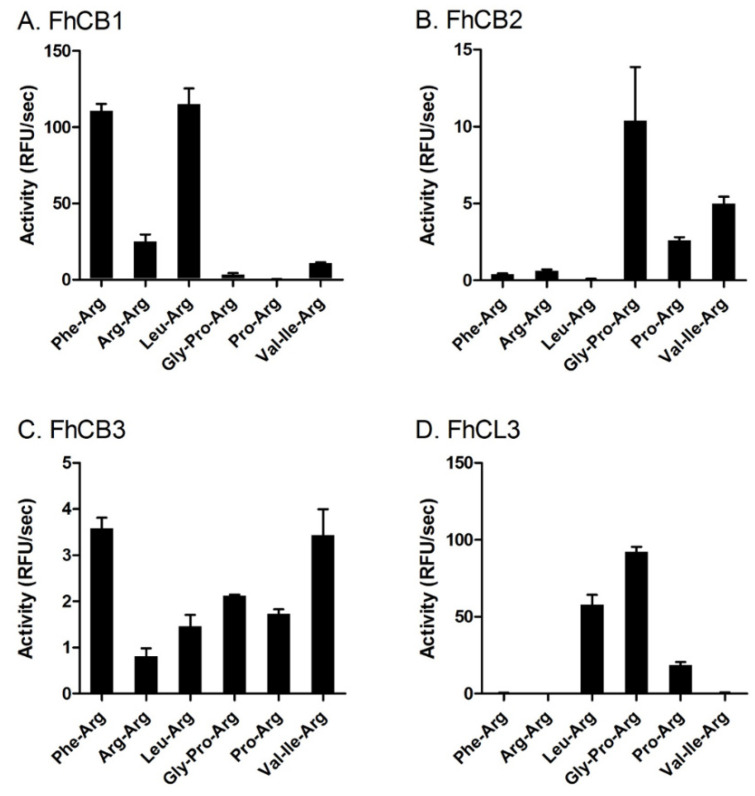
Substrate specificity of FhCB1, FhCB2, FhCB3 and FhCL3. The substrate specificities of each recombinant peptidase, FhCB1 (**A**), FhCB2 (**B**), FhCB3 (**C**) and FhCL3 (**D**), were assessed using six fluorogenic peptide substrates, Z-Phe-Arg-AMC (Phe-Arg), Z-Arg-Arg-AMC (Arg-Arg), Z-Leu-Arg-AMC (Leu-Arg), Z-Gly-Pro-Arg-AMC (Gly-Pro-Arg), Z-Pro-Arg-AMC (Pro-Arg) and Z-Val-Ile-Arg-AMC (Val-Ile-Arg), in 0.1 M C-P buffer with 1 mM DTT at the optimum pH determined for each peptidase (see Figure 2). Enzyme activity is represented as relative fluorescent units (RFU/s). Error bars indicate standard deviation of three replicates performed in two experiments.

The endopeptidase substrate specificity of FhCB1, FhCB2, FhCB3 and FhCL3 was examined using six different N-terminally blocked fluorogenic peptide substrates at the optimum pH for each enzyme. Under these conditions, FhCB1 showed a clear preference for substrates Z-Phe-Arg-AMC and Z-Leu-Arg-AMC (Figure 3A). By contrast, FhCB2 exhibited low activity against these two substrates, while, at the same time, showing a preference for Z-Gly-Pro-Arg-AMC, Z-Pro-Arg-AMC and Z-Val-Ile-Arg-AMC (Figure 3B). FhCB3 displayed a more promiscuous activity, as it hydrolysed all six fluorogenic peptide substrates examined, although it preferred the substrates Z-Phe-Arg-AMC and Z-Val-Ile-Arg-AMC (Figure 3C). Surprisingly, all three FhCBs showed relatively weak activity against Z-Arg-Arg-AMC, a substrate generally considered to show selectivity towards cathepsins B [32,33].

FhCL3 exhibited a unique substrate profile compared to FhCB1, -2 and -3; it effectively hydrolysed Z-Gly-Pro-Arg-AMC (Figure 3D). This peptidase also cleaved Z-Pro-Arg-AMC but with relatively reduced efficiency, highlighting the importance of Gly in the P3 position for binding to the enzyme’s active site. FhCL3 also cleaved Z-Leu-Arg-AMC but not Z-Phe-Arg-AMC.

### 3.4. Kinetic Analyses of the Enzymatic Efficiency of FhCB1, FhCB2, FhCB3 and FhCL3

The comparative kinetic parameters, *K*_M_ and *K*_cat_, of the various enzyme–substrate interactions were determined. The substrates employed for these analyses were the substrates with greater preference for one or more enzymes, as shown in Figure 3: Z-Phe-Arg-AMC (FhCB1, FhCB3), Z-Gly-Pro-Arg-AMC (FhCB2, FhCB3, FhCL3) and Z-Val-Ile-Arg-AMC (FhCB2, FhCB3) (Table 1). Based on the terminology derived by Koshland [34], the catalytic efficiency of the enzyme against each substrate was determined by the ratio *K*_cat/_*K*_M_.

The hydrolysis of Z-Phe-Arg-AMC was observed to be the most efficient for FhCB1 (*K*_cat_/*K*_M_ of 13,596 M^−1^S^−1^), which exhibited a value at least eight times greater than that obtained with FhCB3 (*K*_cat_/*K*_M_ of 1708 M^−1^S^−1^).

The catalytic efficiency of FhCB2 against Z-Gly-Pro-Arg-AMC and Z-Val-Ile-Arg-AMC was similar (*K*_cat_/*K*_M_ of 4933 and 6876 M^−1^S^−1^, respectively) and more efficient than that observed for FhCB3 (*K*_cat_/*K*_M_ of 579 and 2266 M^−1^S^−1^, respectively). For both enzymes, Z-Val-Ile-Arg-AMC was the best peptide substrate.

FhCL3 hydrolysed Z-Gly-Pro-Arg-AMC very effectively with a *K*_cat_/*K*_M_ value of 17,960 M^−1^S^−1^, which is 2.3 and 31 times greater than the ratio obtained with FhCB2 and FhCB3, respectively.

### 3.5. Exo-Carboxypeptidase Activity

**Figure 4:**
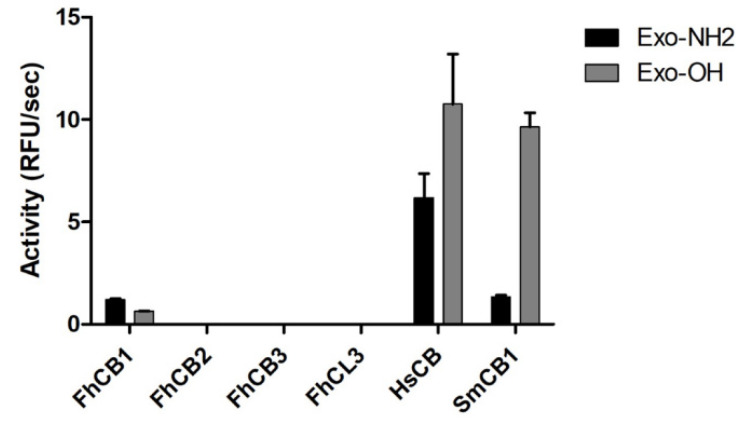
Exo-carboxypeptidase activity of FhCB1, FhCB2, FhCB3 and FhCL3. The exopeptidase activity was examined using two fluorescent substrates, Abz-Phe-Arg-Ala-Lys(Dnp)-OH (Exo-OH) and Abz-Phe-Arg-Ala-Lys(Dnp)-NH_2_ (Exo-NH_2_), where Abz is 2-aminobenzoyl and Dnp is 2,4-dinitrophenyl. The reactions were carried out in 0.1 M C-P buffer, pH 5.0, with a final substrate concentration of 20 μM. The exo-carboxypeptidase activity of the FhCBs and FhCL3 was compared with the activity of the *Schistosoma mansoni* cathepsin B1 (SmCB1) and human cathepsin B (HsCB), represented as relative fluorescent units (RFU/s). Error bars indicate standard deviation of three replicates performed in two experiments.

The exopeptidase activity of the four recombinant peptidases was assayed against two fluorogenic exopeptidase substrates, Abz-Phe-Arg-Ala-Lys(Dnp)-OH (Exo-OH) and Abz-Phe-Arg-Ala-Lys(Dnp)-NH_2_ (Exo-NH_2_), designed to span the S2-S2′ subsites of the active peptidases [35]. Of the four *F. hepatica* cathepsin peptidases assessed, only the recombinant FhCB1 cleaved Exo-OH and Exo-NH_2_; however, this activity was similar to that observed for SmCB1, but both were relatively low compared with the positive human control, HsCB (Figure 4).

### 3.6. Inhibitors Screening against FhCB1, FhCB2, FhCB3 and FhCL3

**Figure 5:**
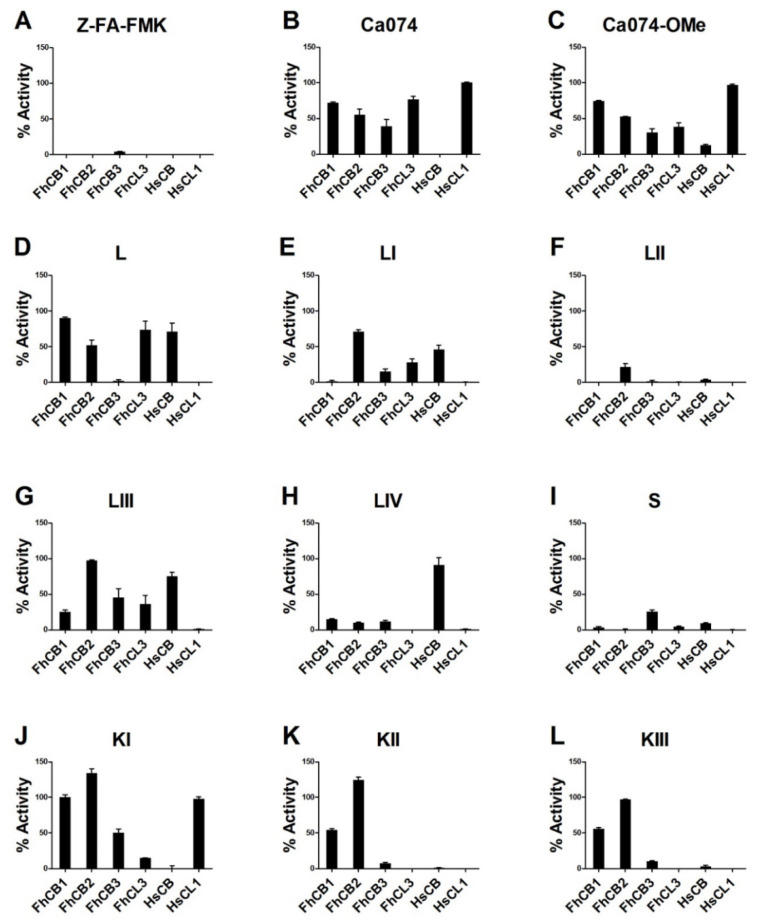
Profile of inhibition of FhCB1, FhCB2, FhCB3 and FhCL3. Commercially available inhibitors of cathepsin B (Ca074 and Ca07a-OMe), cathepsin L (L, L1, LII, LIII and LIV), cathepsin K (KI, KII and KIII), cathepsin S and the broad-spectrum papain peptidase inhibitor Z-Phe-Ala-FMK (**A**–**L**) were used to determine and compare the inhibition constant against recombinants FhCB1, FhCB2, FhCB3, FhCL3, HsCB and HsCL1. Activity is represented as the percentage of enzymatic activity observed by hydrolysis of a fluorogenic quenched peptide substrate following a pre-incubation with inhibitor in relation to the activity of the enzyme alone. Error bars indicate standard deviation of three replicates.

A panel of 12 commercially available cysteine peptidase inhibitors that preferentially target mammalian cathepsin B (Ca074 and Ca074-OMe), cathepsin L (L, L1, LII, LIII and LIV) cathepsin S (S) and cathepsin K (KI, KII and KIII) were screened against the FhCBs, FhCL3 and, as controls, HsCB and HsCL1. The broad-range cysteine peptidase inhibitor Z-Phe-Ala-FMK was also included (Figure 5).

Each *F. hepatica* peptidase displayed a distinct profile against the inhibitors tested. However, it is noteworthy that the epoxysuccinyl cathepsin B inhibitors, Ca074 and Ca074-OMe, which we confirmed to be selective for HsCB compared to HsCL1, were poor inhibitors of all three FhCBs and of FhCL3 (Figure 5). Our studies show that the inhibitory constants (*K*_i_) were relatively high for these inhibitors against all FhCBs (Table 2). However, FhCB1, FhCB2 and FhCB3 were most potently inhibited by Z-Phe-Ala-FMK, LII, LIV and inhibitor S (*K*_i_ = 345 nM). The compounds KII and KIII abrogated the activity of FhCB3, whilst they were only weak inhibitors of FhCB1 and had no effect against FhCB2.

Compared to the FhCBs, the activity of FhCL3 peptidase was potently abrogated by the inhibitor KII (*K*_i_ = 0.7 nM). In addition, Phe-Ala-FMK, LII and S also strongly inhibited FhCL3 (*K*_i_ =15 nM, 8 nM and 35 nM, respectively).

### 3.7. Effect of Cysteine Peptidases Inhibitors on F. hepatica Metacercarial Excystment In Vitro

**Figure 6:**
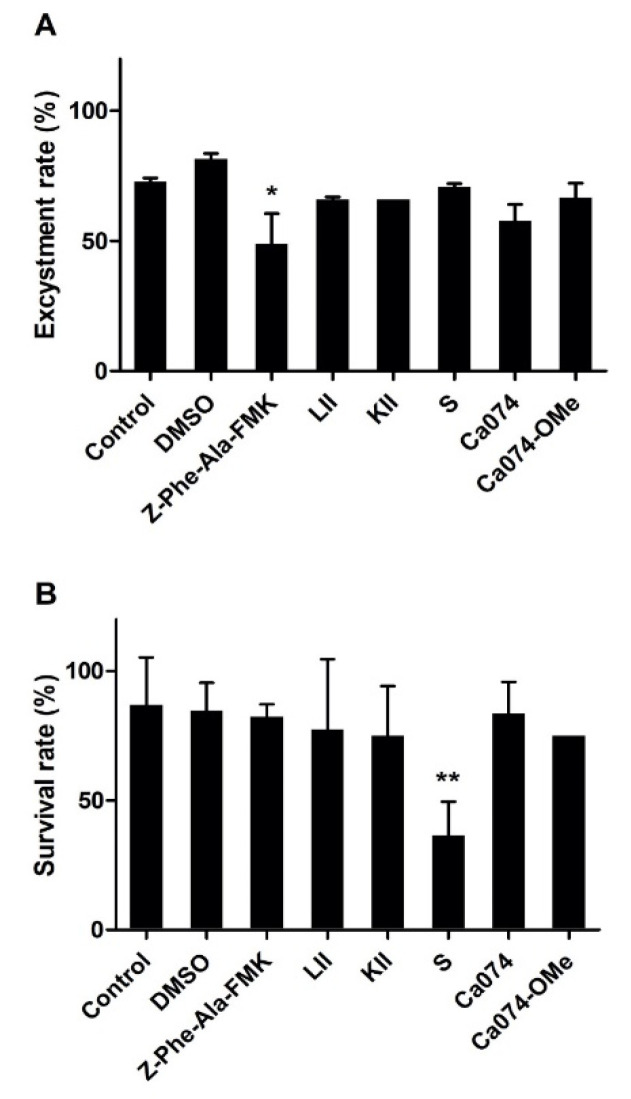
Effect of cysteine proteases inhibitors on metacercariae excystment and NEJ survival. Effects of inhibitors on (**A**) the excystment of *F. hepatica* metacercariae and (**B**) viability of NEJ. (**A**) Fifty metacercariae per well were placed into a 24-well plate and 1 mL of excystment medium supplemented with 100 μM of either inhibitor was added to the well. PBS and dimethylsulfoxide (DMSO, 1:100) were used as controls. (**B**) NEJ viability was assessed by culturing the parasites in 1 mL of culture medium containing 100 μM of the inhibitor at 5% CO_2_ at 37 °C for 24 h. Percentage excystment rate and survival rate were calculated based on the starting number of parasites in each well. Error bars indicate standard deviation of three replicates. * *p* > 0.05; ** *p* > 0.01.

The excystment of *F. hepatica* metacercariae was examined in the presence of the most efficient inhibitors, namely, Phe-Ala-FMK, LII, KII, S, Ca074 and Ca074-OMe, each at a final concentration of 100 μM (Figure 6). In the untreated group (PBS and DMSO control), the average excystment rate of metacercariae was 73–83%. While most of the inhibitor compounds just slightly decreased the *F. hepatica* metacercariae excystment rate, the broad-range inhibitor Z-Phe-Ala-FMK was shown to significantly impact excystment by reducing it by more than 50% (*p* ≤ 0.005) (Figure 6A).

The survival rate of NEJ in culture in the presence of the inhibitors was also examined. A significant effect was only observed with inhibitor S, which reduced the viability of the parasites by ~55% compared to the DMSO group (*p* ≤ 0.001). None of the other inhibitors tested significantly influenced the survival of this stage of the parasite (Figure 6B).

## 4. Discussion

To establish infection in their host, NEJ of *F. hepatica* must migrate from the intestinal lumen, through the intestinal wall and to the liver. During this migration the juvenile parasites encounter several layers of host tissues and various macromolecules that create physical barriers. Immunolocalisation studies using polyclonal antibodies showed that NEJ produce copious amounts of FhCBs and FhCLs by the gastrodermal cells of the parasite gut [36,37,38]. Their secretion from these cells is critical in parasite virulence, as RNAi-induced silencing of these peptidases significantly reduces tissue penetration in vitro [36]. However, it is still unclear whether each peptidase plays similar or distinct roles in virulence. Here, we comprehensively characterised recombinant forms of the four major NEJ peptidases, FhCB1, FhCB2, FhCB3 and FhCL3, and show that they displayed major biochemical differences.

The optimal pH for proteolysis by FhCB1 and FhCB2 was observed at pH 5.0, while that observed for FhCB3 and FhCL3 was closer to neutral, pH 7.0 and pH 6.5, respectively. The optimal pH for activity is often indicative of the physiological pH found at the site of action for a given peptidase; therefore, these results suggest that peptidases function in different places within the parasite or host [28]. Significantly, the digestive tract of *F. hepatica* is maintained at slightly acidic pH, around pH 5.5 [28,39], while the pH of the host intestinal tract is between 6.0 and 7.4 [40]. This could suggest that FhCB1 and FhCB2 function predominantly in the parasite gut lumen, while FhCB3 and FhCL3 play a more prominent role in the penetration of the host tissues. Indeed, FhCB3 and FhCL3 are the most prominent peptidases found in the ES of juvenile parasites maintained in vitro [10,37,41,42].

FhCL3, FhCB2 and, to a lesser extent, FhCB3, showed selectivity toward cleavage of the substrate, Z-Gly-Pro-Arg-AMC, that is indicative of collagenolytic activity. Orthologous peptidases of FhCB2 and FhCB3 from *F. gigantica*, FgCB2 and FgCB3, respectively, were also shown to digest type I collagen [43]. We have previously reported that FhCL3 exhibits a similar substrate specificity to the collagenolytic enzyme cathepsin K and likely aids in the degradation of host tissue in order to facilitate migration of parasite NEJ from the intestinal lumen to the liver [44]. This present study is the first to indicate that NEJ FhCB2 and FhCB3 may function in concert with FhCL3 to degrade the interstitial matrix of tissues to facilitate the parasite’s migration through the intestinal wall. However, kinetics studies show that FhCL3 displays the greatest activity on Z-Gly-Pro-Arg-AMC and its performance constant was found to be 2.3 and 31 times greater than FhCB2 and FhCB3, respectively.

By contrast to the other three peptidases, FhCB1 showed little activity towards substrates containing a Pro residue at the P2 position, suggesting that this peptidase is not directly involved with collagen degradation. However, FhCB1 also differed in having very low activity against Z-Arg-Arg-AMC, a substrate typically cleaved by cathepsins B. Greater activity was observed for FhCB1 against substrates containing the peptides Z-Phe-Arg-AMC and Z-Leu-Arg-AMC, indicating a preference for hydrophobic P2 residues, which is more typical of cathepsin L endopeptidase activity [44]. This unique property may suggest a distinctive function worth elucidating in the future.

The S2 subsite of the active site of cathepsin B peptidases are of prime importance for holding substrates in position; these are largely conserved in cathepsin B peptidases (Supplementary Figure S1). Of particular note is the residue Glu316, which, in SmCB1, is found at the bottom of the S2 subsite and is shown to interact directly with the substrate/inhibitor P2 residue and change its orientation within the S2 subsite (Glu246 in SmCB) [24]. However, in FhCB1 and FhCB3, this residue is replaced by the smaller hydrophobic Ile and basic Arg, respectively, while FhCB2 retains the acidic Glu. This single residue difference has a significant impact on the substrate specificities of the enzymes as, for example, when Glu316 was replaced by Gln, in rat cathepsin B, it significantly decreased its ability to accommodate an Arg residue in its S2 site [45]. Using the substrate Z-Phe-Arg-AMC, we found that FhCB1 had a value of *K*_cat_ (substrate turnover) more than 20 times higher than that of FhCB3, while this substrate was not cleaved by FhCB2. On the other hand, both FhCB2 and FhCB3 preferably cleaved Z-Val-Ile-Arg-AMC over all other substrates examined, whereas this was a poor substrate for FhCB1. Collectively, these data indicate major difference in the substrate specificity of the three *F. hepatica* cathepsin B peptidases.

Cathepsin B peptidases are uniquely characterised by having a flexible loop structure, termed the occluding loop, which is located at the apical region of the active site cleft and confers these enzymes with unique carboxy-peptidyl-dipeptidase activity. This activity brings about the removal of two amino acid residues from the carboxy-terminus of protein substrates [24,35,46,47]. Deletion of the occluding loop sequence not only obliterates this exopeptidase activity but also increases the endopeptidase activity of the enzyme [48]. This exopeptidase activity is facilitated by the presence of two neighbouring His residues in the occluding loop, at positions 110 and 111 (*S. mansoni* mature cathepsin B1 enzyme numbering in Jílková et al. [24]; Supplemental material Figure S1), which provides a positively charged anchor for the C-terminal carboxy group of the substrate. However, only His110 is thought to be critical for exopeptidase activity, while His111 increases the positive charge on the loop and increases the binding potential. To hold the occluding loop in an appropriate conformation for exopeptidase activity, in human cathepsin B, the His110 forms a salt-bridge interaction with Asp93, which is strengthened by the presence of His181 [35].

Sequence alignments show that FhCB1, FhCB2 and FhCB3 possess an occluding loop, but, while these retain His110, they each lack His 111 (Supplemental Material Figure S1). Absence of this residue has been previously shown to deplete the carboxydipeptidyl activity of FhCB2 [15], while *F. gigantica* cathepsin B5, which possesses both His110 and His111, exhibits exopeptidase activity [49]. Our studies add support to these previous reports by showing that FhCB2 and FhCB3 do not possess carboxydipeptidyl activity, while FhCB1 exhibits low but significant exopeptidase activity. Of further interest is the fact that the compounds Ca074 and Ca074-OMe, that specifically bind and inhibit mammalian cathepsin B by forming H-bonds with His110 and His111 on the occluding loop [21], were very poor inhibitors of the *F. hepatica* cathepsins B.

Interestingly, we found that the occluding loops of the *F. hepatica* and *S. mansoni* cathepsins B were not highly conserved. This region only shares 11–23% sequence identity between the FhCBs, whereas it is generally conserved in cathepsins B from mammalian species [24]. This unusual non-conserved region could contribute to the lack of carboxydipeptidyl activity found in the *F. hepatica* cathepsins B and/or could confer unique, so far undisclosed, exopeptidase activities.

A screen of commercially available cathepsin inhibitors revealed that the three FhCBs and FhCL3 exhibited distinct inhibition profiles. While the five cathepsin L inhibitors (L, LI, LII, LIII and LIV) showed specificity for HsCL1 over HsCBs, they exhibited high variability against the FhCBs and FhCL3, again emphasising the difference between the parasite enzymes and their mammalian hosts. For example, the epoxysuccinyl peptide inhibitor, L, which did not inhibit FhCB1 or FhCB2, significantly reduced FhCB3 and FhCL3 activity, whereas L1, containing a Phe-Phe peptide, was the most effective at blocking FhCB1 and FhCB3 activity. LII, which contains the dipeptide Phe-Tyr, was the most effective at reducing the enzymatic activity of all *F. hepatica* peptidases but this compound also inhibited human cathepsin L and B demonstrating a potent but less specific interaction of this inhibitor with the parasite and host enzymes.

The mammalian cathepsin K inhibitors (KI, KII and KIII) were generally poor inhibitors of FhCB1 and FhCB2 but reduced FhCB3 and FhCL3 activity. This observation is in agreement with our earlier finding that FhCL3 exhibits cathepsin K-like collagenase activity [44] and further implies that FhCB3, which can also cleave the substrate Z-Gly-Pro-Arg-AMC, may collaborate with FhCL3 to perform this activity in vivo. However, despite the fact that FhCB2 can cleave Z-Gly-Pro-Arg-AMC, it was not inhibited by the KI, KII or KIII compound.

Detailed kinetics studies were performed with six inhibitors and confirmed the poor inhibition of all four parasite enzymes with the inhibitors Ca074 and Ca074-OMe. In general, FhCB1 and FhCL3 were more susceptible to inhibition by cysteine peptidase inhibitors than FhCB2 and FhCB3. Nevertheless, we found that the cathepsin L inhibitor, LII and the broad range inhibitors Z-Phe-Ala-FMK were extremely potent inhibitors of FhCB1 and FhCL3 with *K*_i_ values in the low nM range, <15 nM. The cathepsin S inhibitor, S, a Phe-Leu dipeptide with a keto-aldehyde (COCHO) group, which has reported *K*_i_ values for inhibition of human cathepsins S and B of 0.185 nM and 76 nM, respectively, ref. [50] was the next best inhibitor (*K*_i_ values of 14 nM and 35 nM for FhCB1 and FhCL3, respectively).

Our NEJ excystment experiments revealed that the majority of cysteine protease inhibitors examined at a 100 μM final concentration had little effect on the ability of the fluke to emerge from the cysts. The only compound to show significant inhibition of excystment was Z-Phe-Ala-FMK. Previous studies from our laboratory have shown that a final concentration of 1 mM of this compound completely prevented metacercariae excystment [10]. This suggests that the inhibitors do not readily pass through the metacercarial cyst walls and supports the suggestion by of Dixon and Mercer [51] that the metacercarial cyst walls are generally impenetrable to small compounds.

When NEJ were cultured for 24 h with each inhibitor, we observed that most of the cysteine peptidase inhibitors did not elicit a significant effect on parasite viability. Our data differ from those of Beckham et al. [15], who reported that the cathepsin B inhibitor, Ca074-OMe, had a sub-lethal effect on NEJ cultured in a concentration of 6.25 μM. In our studies, we found that the inhibitor S, an effective inhibitor of FhCBs and FhCL3, was the only effective flukicide against NEJ within 24 h of in vitro culture. The compound S belongs to a group of inhibitors known as alpha-keto-beta-aldehydes that contain two highly electrophilic carbons (α and β) in their C-terminal group that facilitate strong and irreversible binding to the cathepsin cysteine peptidases [52,53]. However, the potency and indiscriminate nature of alpha-keto-beta-aldehydes in protease inhibition (since they also inhibit serine peptidases) may have implications for their toxicity to host biology and potentially prevent their use as therapeutics.

In summary, we undertook a series of experiments on enzyme–substrate/inhibitor kinetics and revealed distinct differences in the specificity of the NEJ FhCB1, FhCB2, FhCB3 and FhCL3. This variation provides evidence for distinct and over-lapping functional roles of the peptidases that would allow the parasite to degrade a multitude of macromolecular substrates. Investigations into the digestion of protein substrates from mammalian hosts susceptible to *Fasciola* infection have shown that NEJ proteinases catalyse the hydrolysis of gelatin [54,55], collagen [43,44,56], fibronectin [57], serum albumin [12], immunoglobulins [58] and haemoglobin [28]. Collectively, the data support our hypothesis that peptidases secreted by juvenile flukes act together as a powerful hydrolytic mix to facilitate tissue migration through digestion of host tissues, which would otherwise act as physical barriers to invasion, with complementary functions in feeding and immune evasion. The blocking of the peptidase activity in vitro did not affect parasite viability, but this may be because the enzymes predominantly function extra-corporeally. However, we have previously shown that RNAi-mediated knockdown of FhCL and FhCB expression in NEJ did prevent the parasites’ migration through the gut wall in culture [36]. Hence, the blocking of their activities in vivo by chemical or immunological means could still offer a route towards developing new anti-fluke treatments.

## Figures and Tables

**Table 1:** Kinetics of cleavage of fluorogenic peptide substrates by recombinant FhCBs and FhCL3, represented by *K*_M_ (μM), *K*_cat_ (s^−1^) and *K*_cat_/*K*_M_ (M^−1^S^−1^).

Peptidase		Z-Phe-Arg-AMC	Z-Gly-Pro-Arg-AMC	Z-Val-Ile-Arg-AMC
FhCB1	*K*_M_	69 ± 5 *		
	*K*_cat_	1.0 ± 0.2	-	-
	*K*_cat_/*K*_M_	13,596		
FhCB2	*K*_M_		52 ± 4	71 ± 7
	*K*_cat_	-	0.4 ± 0.001	0.5 ± 0.004
	*K*_cat_/*K*_M_		4933	6876
FhCB3	*K*_M_	27 ± 2	40 ± 4	42 ± 4
	*K*_cat_	0.05 ± 0.007	0.02 ± 0.002	0.1 ± 0.004
	*K*_cat_/*K*_M_	1708	579	2266
FhCL3	*K*_M_		27 ± 2	
	*K*_cat_	-	0.5 ± 0.07	-
	*K*_cat_/*K*_M_		17,960	

* Values are represented ± standard deviation.

**Table 2:** Inhibition constants of various papain-like peptidase inhibitors against recombinants FhCB1, FhCB2, FhCB3 and FhCL3 represented by the inhibition constant *K*_i_ (nM ± SEM).

Inhibitor	FhCB1	FhCB2	FhCB3	FhCL3
Z-Phe-Ala-FMK	3 ± 0.20	904 ± 271	246 ± 55.74	15 ± 5.95
LII	7 ± 1.25	1840 ± 77	211 ± 34.84	8 ± 0.60
KII	466 ± 40.59	NI *	227 ± 28.32	0.7 ± 0.19
S	14 ± 2.42	345 ± 18.6	696 ± 94.80	35 ± 2.4
Ca074	7317 ± 934	5930 ± 567.1	540 ± 45.22	603 ± 133.3
Ca074ME	5500 ± 1187	3892 ± 299	785 ± 130.4	249 ± 35.48

* NI—no inhibition.

## Data Availability

No new data were created or analysed in this study. Data sharing is not applicable to this article.

## Supplementary Materials

The following are available online at https://www.mdpi.com/article/10.3390/ani11123495/s1, Figure S1: Alignment of cathepsin B peptidases. Figure S2: Structure of the commercial cathepsin inhibitors.

## References

[1] Mazeri S., Rydevik G., Handel I., Bronsvoort B.M.D., Sargison N. (2017). Estimation of the impact of *Fasciola hepatica* infection on time taken for UK beef cattle to reach slaughter weight. Sci. Rep..

[2] Sripa B. (2012). Global burden of food-borne trematodiasis. Lancet Infect. Dis..

[3] Fürst T., Keiser J., Utzinger J. (2012). Global burden of human food-borne trematodiasis: A systematic review and meta-analysis. Lancet Infect. Dis..

[4] Caravedo M.A., Cabada M.M. (2020). Human fascioliasis: Current epidemiological status and strategies for diagnosis, treatment, and control. Res. Rep. Trop. Med..

[5] Gonzalez-Miguel J., Becerro-Recio D., Siles-Lucas M. (2021). Insights into *Fasciola hepatica* juveniles: Crossing the fasciolosis rubicon. Trends Parasitol..

[6] Boray J.C. (1969). Experimental fascioliasis in Australia. Adv. Parasitol..

[7] Graczyk T.K., Fried B., Dalton J.P. (1999). Development of *Fasciola hepatica* in the intermediate host. Fasciolosis.

[8] Mas-Coma S., Valero M.A., Bargues M.D. (2014). Fascioliasis. Adv. Exp. Med. Biol..

[9] Cwiklinski K., Donnelly S., Drysdale O., Jewhurst H., Smith D., De Marco Verissimo C., Pritsch I.C., O’Neill S., Dalton J.P., Robinson M.W. (2019). The cathepsin-like cysteine peptidases of trematodes of the genus *Fasciola*. Adv. Parasitol..

[10] Robinson M.W., Menon R., Donnelly S.M., Dalton J.P., Ranganathan S. (2009). An integrated transcriptomics and proteomics analysis of the secretome of the helminth pathogen *Fasciola hepatica*: Proteins associated with invasion and infection of the mammalian host. Mol. Cell. Proteom..

[11] Di Maggio L.S., Tirloni L., Pinto A.F.M., Diedrich J.K., Yates J.R., Carmona C., Berasain P., da Silva Vaz I. (2019). A proteomic comparison of excretion/secretion products in *Fasciola hepatica* newly excysted juveniles (NEJ) derived from *Lymnaea viatrix* or *Pseudosuccinea columella*. Exp. Parasitol..

[12] Wilson L.R., Good R.T., Panaccio M., Wijffels G.L., Sandeman R.M., Spithill T.W. (1998). *Fasciola hepatica*: Characterization and cloning of the major cathepsin B protease secreted by newly excysted juvenile liver fluke. Exp. Parasitol..

[13] Law R.H., Smooker P.M., Irving J.A., Piedrafita D., Ponting R., Kennedy N.J., Whisstock J.C., Pike R.N., Spithill T.W. (2003). Cloning and expression of the major secreted cathepsin B-like protein from juvenile *Fasciola hepatica* and analysis of immunogenicity following liver fluke infection. Infect. Immun..

[14] Beckham S.A., Law R.H., Smooker P.M., Quinsey N.S., Caffrey C.R., McKerrow J.H., Pike R.N., Spithill T.W. (2006). Production and processing of a recombinant *Fasciola hepatica* cathepsin B-like enzyme (FhcatB1) reveals potential processing mechanisms in the parasite. Biol. Chem..

[15] Beckham S.A., Piedrafita D., Phillips C.I., Samarawickrema N., Law R.H., Smooker P.M., Quinsey N.S., Irving J.A., Greenwood D., Verhelst S.H. (2009). A major cathepsin B protease from the liver fluke *Fasciola hepatica* has atypical active site features and a potential role in the digestive tract of newly excysted juvenile parasites. Int. J. Biochem. Cell Biol..

[16] Corvo I., Cancela M., Cappetta M., Pi-Denis N., Tort J.F., Roche L. (2009). The major cathepsin L secreted by the invasive juvenile *Fasciola hepatica* prefers proline in the S2 subsite and can cleave collagen. Mol. Biochem. Parasitol..

[17] Robinson M.W., Donnelly S., Hutchinson A.T., To J., Taylor N.L., Norton R.S., Perugini M.A., Dalton J.P. (2011). A family of helminth molecules that modulate innate cell responses via molecular mimicry of host antimicrobial peptides. PLoS Pathog..

[18] Turk D., Guncar G., Podobnik M., Turk B. (1998). Revised definition of substrate binding sites of papain-like cysteine proteases. Biol. Chem..

[19] Vernet T., Tessier D.C., Chatellier J., Plouffe C., Lee T.S., Thomas D.Y., Storer A.C., Ménard R. (1995). Structural and functional roles of asparagine 175 in the cysteine protease papain. J. Biol. Chem..

[20] Fujishima A., Imai Y., Nomura T., Fujisawa Y., Yamamoto Y., Sugawara T. (1997). The crystal structure of human cathepsin L complexed with E-64. FEBS Lett..

[21] Yamamoto A., Hara T., Tomoo K., Ishida T., Fujii T., Hata Y., Murata M., Kitamura K. (1997). Binding mode of CA074, a specific irreversible inhibitor, to bovine cathepsin B as determined by X-ray crystal analysis of the complex. J. Biochem..

[22] Jia Z., Hasnain S., Hirama T., Lee X., Mort J.S., To R., Huber C.P. (1995). Crystal structures of recombinant rat cathepsin B and a cathepsin B-inhibitor complex. Implications for structure-based inhibitor design. J. Biol. Chem..

[23] Turk D., Podobnik M., Kuhelj R., Dolinar M., Turk V. (1996). Crystal structures of human procathepsin B at 3.2 and 3.3 Angstroms resolution reveal an interaction motif between a papain-like cysteine protease and its propeptide. FEBS Lett..

[24] Jílková A., Řezáčová P., Lepšík M., Horn M., Váchová J., Fanfrlík J., Brynda J., McKerrow J.H., Caffrey C.R., Mareš M. (2011). Structural basis for inhibition of cathepsin B drug target from the human blood fluke, Schistosoma mansoni. J. Biol. Chem..

[25] Pritsch I.C., Tikhonova I.G., Jewhurst H.L., Drysdale O., Cwiklinski K., Molento M.B., Dalton J.P., Verissimo C.M. (2020). Regulation of the *Fasciola hepatica* newly excysted juvenile cathepsin L3 (FhCL3) by its propeptide: A proposed ‘clamp-like’ mechanism of binding and inhibition. BMC Mol. Cell Biol..

[26] Groves M.R., Coulombe R., Jenkins J., Cygler M. (1998). Structural basis for specificity of papain-like cysteine protease proregions toward their cognate enzymes. Proteins.

[27] Stack C.M., Caffrey C.R., Donnelly S.M., Seshaadri A., Lowther J., Tort J.F., Collins P.R., Robinson M.W., Xu W., McKerrow J.H. (2008). Structural and functional relationships in the virulence-associated cathepsin L proteases of the parasitic liver fluke, *Fasciola hepatica*. J. Biol. Chem..

[28] Lowther J., Robinson M.W., Donnelly S.M., Xu W., Stack C.M., Matthews J.M., Dalton J.P. (2009). The importance of pH in regulating the function of the *Fasciola hepatica* cathepsin L1 cysteine protease. PLoS Negl. Trop. Dis..

[29] Collins P.R., Stack C.M., O’Neill S.M., Doyle S., Ryan T., Brennan G.P., Mousley A., Stewart M., Maule A.G., Dalton J.P. (2004). Cathepsin L1, the major protease involved in liver fluke (*Fasciola hepatica*) virulence: Propetide cleavage sites and autoactivation of the zymogen secreted from gastrodermal cells. J. Biol. Chem..

[30] Copeland R.A. (2000). Enzymes: A Practical Introduction to Structure, Mechanism, and Data Analysis.

[31] Piedrafita D., Parsons J.C., Sandeman R.M., Wood P.R., Estuningsih S.E., Partoutomo S., Spithill T.W. (2001). Antibody-dependent cell-mediated cytotoxicity to newly excysted juvenile *Fasciola hepatica* in vitro is mediated by reactive nitrogen intermediates. Parasite Immunol..

[32] Barrett A.J., Kirschke H. (1981). Cathepsin B, cathepsin H, and cathepsin L. Methods Enzymol..

[33] Barrett A.J. (1980). Fluorimetric assays for cathepsin B and cathepsin H with methylcoumarylamide substrates. Biochem. J..

[34] Koshland D.E. (2002). The Application and Usefulness of the Ratio kcat/KM. Bioorg. Chem..

[35] Krupa J.C., Hasnain S., Nägler D.K., Ménard R., Mort J.S. (2002). S2′ substrate specificity and the role of His110 and His111 in the exopeptidase activity of human cathepsin B. Biochem. J..

[36] McGonigle L., Mousley A., Marks N.J., Brennan G.P., Dalton J.P., Spithill T.W., Day T.A., Maule A.G. (2008). The silencing of cysteine proteases in *Fasciola hepatica* newly excysted juveniles using RNA interference reduces gut penetration. Int. J. Parasitol..

[37] Cwiklinski K., Jewhurst H., McVeigh P., Barbour T., Maule A.G., Tort J., O’Neill S.M., Robinson M.W., Donnelly S., Dalton J.P. (2018). Infection by the helminth parasite *Fasciola hepatica* requires rapid regulation of metabolic, virulence, and invasive factors to adjust to its mammalian host. Mol. Cell. Proteom..

[38] Creaney J., Wilson L., Dosen M., Sandeman R.M., Spithill T.W., Parsons J.C. (1996). *Fasciola hepatica*: Irradiation-induced alterations in carbohydrate and cathepsin-B protease expression in newly excysted juvenile liver fluke. Exp. Parasitol..

[39] Stack C.M., Donnelly S., Lowther J., Xu W., Collins P.R., Brinen L.S., Dalton J.P. (2007). The major secreted cathepsin L1 protease of the liver fluke, *Fasciola hepatica*: A Leu-12 to Pro-12 replacement in the nonconserved C-terminal region of the prosegment prevents complete enzyme autoactivation and allows definition of the molecular events in prosegment removal. J. Biol. Chem..

[40] Fallingborg J. (1999). Intraluminal pH of the human gastrointestinal tract. Dan. Med. Bull..

[41] Morphew R.M., Wright H.A., Lacourse E.J., Porter J., Barrett J., Woods D.J., Brophy P.M. (2011). Towards delineating functions within the fasciola secreted cathepsin l protease family by integrating in vivo based sub-proteomics and phylogenetics. PLoS Negl. Trop. Dis..

[42] Di Maggio L.S., Tirloni L., Pinto A.F., Diedrich J.K., Yates Iii J.R., Benavides U., Carmona C., da Silva Vaz I., Berasain P. (2016). Across intra-mammalian stages of the liver fluke *Fasciola hepatica*: A proteomic study. Sci. Rep..

[43] Chantree P., Wanichanon C., Phatsara M., Meemon K., Sobhon P. (2012). Characterization and expression of cathepsin B2 in Fasciola gigantica. Exp. Parasitol..

[44] Corvo I., O’Donoghue A.J., Pastro L., Pi-Denis N., Eroy-Reveles A., Roche L., McKerrow J.H., Dalton J.P., Craik C.S., Caffrey C.R. (2013). Dissecting the active site of the collagenolytic cathepsin L3 protease of the invasive stage of *Fasciola hepatica*. PLoS Negl. Trop. Dis..

[45] Hasnain S., Hirama T., Huber C.P., Mason P., Mort J.S. (1993). Characterization of cathepsin B specificity by site-directed mutagenesis. Importance of Glu245 in the S2-P2 specificity for arginine and its role in transition state stabilization. J. Biol. Chem..

[46] Aronson N.N., Barrett A.J. (1978). The specificity of cathepsin B. Hydrolysis of glucagon at the C-terminus by a peptidyldipeptidase mechanism. Biochem. J..

[47] Musil D., Zucic D., Turk D., Engh R.A., Mayr I., Huber R., Popovic T., Turk V., Towatari T., Katunuma N. (1991). The refined 2.15 A X-ray crystal structure of human liver cathepsin B: The structural basis for its specificity. EMBO J..

[48] Illy C., Quraishi O., Wang J., Purisima E., Vernet T., Mort J.S. (1997). Role of the occluding loop in cathepsin B activity. J. Biol. Chem..

[49] Siricoon S., Vichasri Grams S., Lertwongvisarn K., Abdullohfakeeyah M., Smooker P.M., Grams R. (2015). Fasciola gigantica cathepsin B5 is an acidic endo- and exopeptidase of the immature and mature parasite. Biochimie.

[50] Walker B., Lynas J.F., Meighan M.A., Brömme D. (2000). Evaluation of dipeptide alpha-keto-beta-aldehydes as new inhibitors of cathepsin S. Biochem. Biophys. Res. Commun..

[51] Dixon K., Mercer E. (1964). The fine structure of the cyst wall of the metacercaria of *Fasciola hepatica*. J. Cell Sci..

[52] Lynas J.F., Hawthorne S.J., Walker B. (2000). Development of peptidyl alpha-keto-beta-aldehydes as new inhibitors of cathepsin L—Comparisons of potency and selectivity profiles with cathepsin B. Bioorg. Med. Chem. Lett..

[53] Lynas J.F., Martin S.L., Walker B. (2001). Synthesis and kinetic evaluation of peptide alpha-keto-beta-aldehyde-based inhibitors of trypsin-like serine proteases. J. Pharm. Pharmacol..

[54] Thorsell W., Bjoerkman N. (1965). Morphological and biochemical studies on absorption and secretion in the alimentary tract of *Fasciola hepatica* L.. J. Parasitol..

[55] Dalton J.P., Heffernan M. (1989). Thiol proteases released in vitro by *Fasciola hepatica*. Mol. Biochem. Parasitol..

[56] Howell R.M. (1966). Collagenase activity of immature *Fasciola hepatica*. Nature.

[57] Sethadavit M., Meemon K., Jardim A., Spithill T.W., Sobhon P. (2009). Identification, expression and immunolocalization of cathepsin B3, a stage-specific antigen expressed by juvenile *Fasciola gigantica*. Acta Trop..

[58] Chapman C.B., Mitchell G.F. (1982). Proteolytic cleavage of immunoglobulin by enzymes released by *Fasciola hepatica*. Vet. Parasitol..

