# Supplementary material for: The Zoonotic Helminth Parasite Fasciola hepatica: Virulence-Associated Cathepsin B and Cathepsin L Cysteine Peptidases Secreted by Infective Newly Excysted Juveniles (NEJ)
Source: Animals (Basel). 2021 Dec 8;11(12):3495. doi: 10.3390/ani11123495 (PMC8698070; doi:10.3390/ani11123495)

The Zoonotic Helminth Parasite *Fasciola hepatica:* Virulence-Associated Cathepsin B and Cathepsin L Cysteine Peptidases Secreted by Infective Newly Excysted Juveniles (NEJ)

Tara Barbour [1], Krystyna Cwiklinski [1,2], Richard Lalor [2], John Pius Dalton [1,2] and Carolina De Marco Verissimo [1,2,*]

Figure 1A. Original SDS-PAGE relating to Fig 1A. The grey box obscures bands not pertinent to this study.

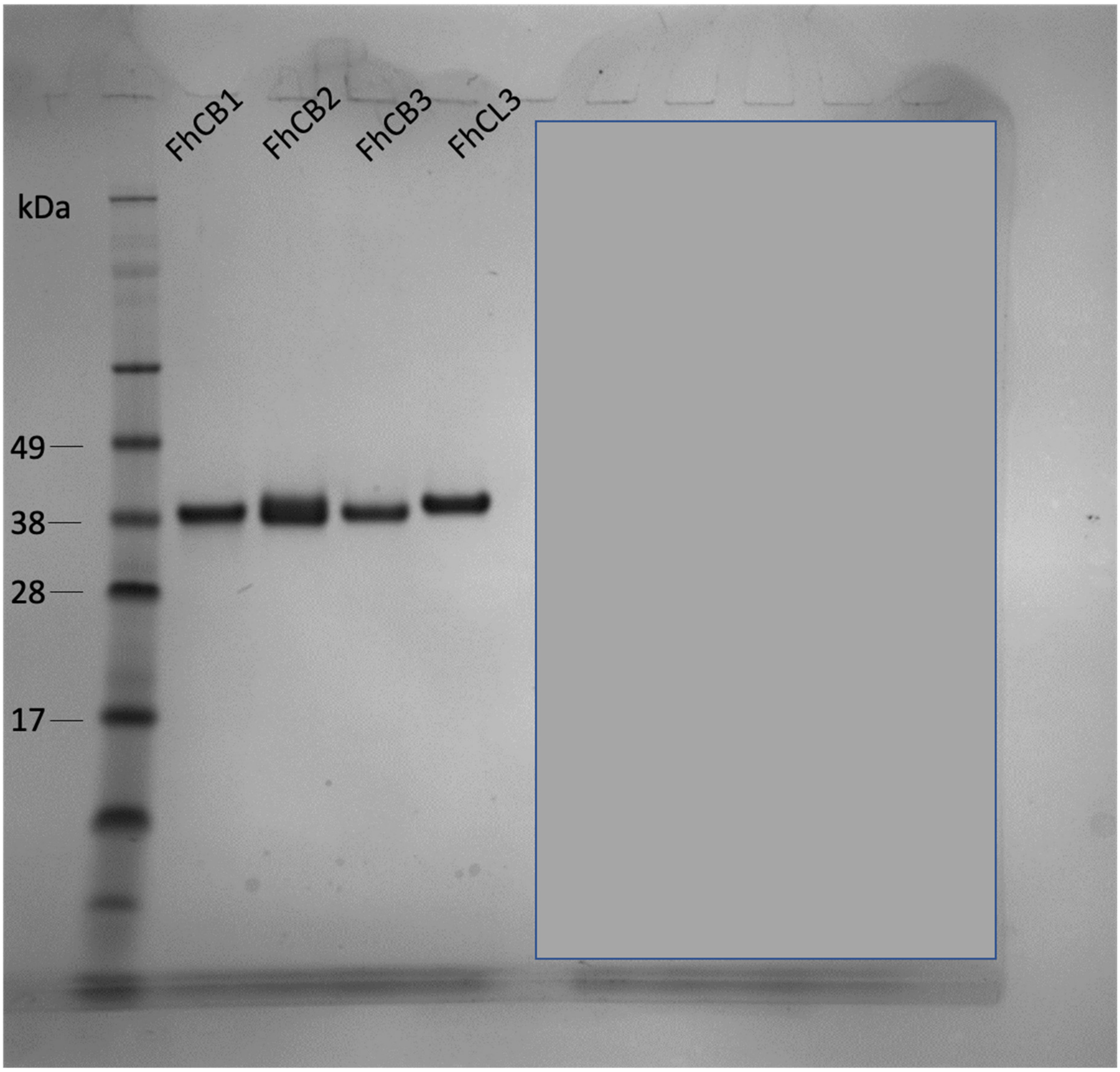

Figure 1B. Original SDS-PAGE relating to Fig 1B. The grey box obscures bands not pertinent to this study.

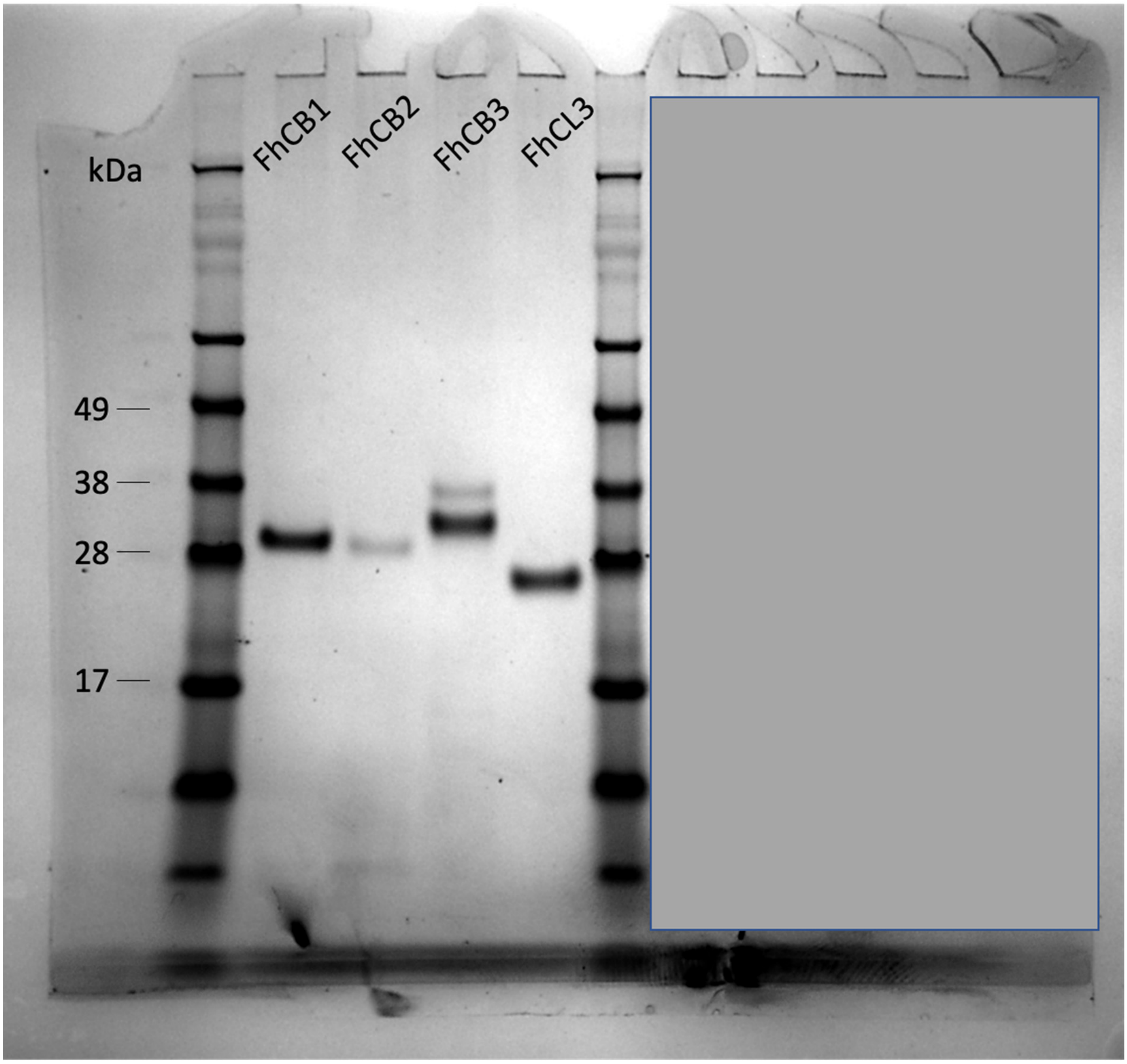

Supplement: Supplementary file 1 [file animals-11-03495-s001.zip › Animals- 1443775- Original gels images.pdf]
