# Supplementary material for: The Zoonotic Helminth Parasite Fasciola hepatica: Virulence-Associated Cathepsin B and Cathepsin L Cysteine Peptidases Secreted by Infective Newly Excysted Juveniles (NEJ)
Source: Animals (Basel). 2021 Dec 8;11(12):3495. doi: 10.3390/ani11123495 (PMC8698070; doi:10.3390/ani11123495)
Supplement: Supplementary file 1 [file animals-11-03495-s001.zip › Supplementary Fig 1.pdf]

A.

```
SmCB1      MLTSILCIASLITFLEAHISVKNEKFEPLSDDIISYINEHPNAGWRAEKSNRFHSLDDAR
B1         ------------VVVQAAPNERKPQFEPFSDELIHYINEKSGASWKAAPSSRFINIEHFK
B2         ----------------------KPQFEPFSDELIRFVNEESGASWKAARSTRFSNVDHFK
B3         -MSWLLIFAA--IVVAQAKPNYKRQFEPFSDELIHYINEESGASWKAAPSTRFNNIDQVK
                                 : :***:**::* ::**.  *.*:*  *.** .::. :

SmCB1      IQMGARREEPDLRRTRRPTVDHNDWNVEIPSSFDSRKKWPRCKSIATIRDQSRCGSCWAF
B1         QHLGLLEETPEERQTRRPTVRYNVSENDLPESFDAREKWPLCRSIRQIPDQSSCGSCWAV
B2         LHLGALSETPEERNALRPTIKHDISKNDLPESFDARSQWPQCWTISEIRDQASCGSCWAT
B3         QNLGVLEETPEDRNTQRQTVRYSVSENDLPESFDARQKWANCPSISEIRDQSSCSSCWAV
            .:*   * *: *.: * *: :.  : ::*.***:*.:*  * :*  * **: *.****

SmCB1      GAVEAMSDRSCIQSGGKQNVELSAVDLLSCCESCGLGCEGGILGPAWDYWVKEGIVTGSS
B1         AGVGAMSDRVCIHSNGMMQPELSAIDLVSCCSYCGNGCQGGSPPAAWDYWWRNGIVTGGT
B2         AAASAMSDRVCIHSNGQMRPRLAAADPLSCCTYCGQGCRGGYPPKAWDYWMREGIVTGGT
B3         SSASAITDRICIHSNGQKKPRLSAIDIVSCCAYCGYGCNGGIPAMSWDYWTREGVVTGGT
           ... *::** **:* *  . .*:* * :***  ** **.**    :**** ::*:***.:

                           Occluding loop
SmCB1      KENHTGCEPYPFPKCEHHT-KGKYPPCGSKIYKTPRCKQTCQKKYKTPYTQDKHRGKSSY
B1         LENPTGCLPYPFPQCRHPGSRSQLNPCPRYIYPTPSCYPYCQAGYDKTYEQDKVYGKTSY
B2         WENRTGCQPWMFTKCDHVGDSRKYSRCPHYTYPTPPCARACQTGYNKTYEQDKFYGNSSY
B3         LENPTGCLPYPFPKCSHGVVTPGLPPCPRDIYPTPKCEKKCHAGYNKTYEQDKVKGKSSY
            ** *** *: * :* *         *    * ** *   *:  *.. * ***  *::**

SmCB1      NVKNDEKAIQKEIMKYGPVEAGFTVYEDFLNYKSGIYKHITGETLGGHAIRIIGWGVENK
B1         NVDRHEYTIMEEIMKNGPVEAGFIVYTDFAVYKSGIYHHVSGRYAGKHAIRIIGWGVENG
B2         NVGEHESYIMQEIMKNGPVEVTFAIFQDFGVYRSGIYHHVAGKFIGRHAVRMIGWGVENG
B3         NVGEQETDIMMEIMKNGPVDGIFYMFEDFLVYKSGIYHYTTGRLVGGHAIRVIGWGVENG
           ** ..*  *  **** ***:  * :: **  *:****:: :*.  * **:*:*******

SmCB1      TPYWLIANSWNEDWGENGYFRIVRGRDECSIESEVTAGRIN---------
B1         VNYWLTANSWNVGWGENGYFRILRGTDECRIESIVVAGMPRLQKNITNHH
B2         VNYWLMANSWNEEWGENGYFRMVRGRNECGIESEVVAGMPRL--------
B3         VKYWLIANSWNEGWGEKGYFRMRRGNNECGIEARINAGLPS---------
           . *** *****  ***:****: ** :** **: : **
```

B.

Position	FhCB1	FhCB2	FhCB3	FhCL3
75	S	Y	I	W
76	P	P	P	M
175	F	F	F	V
199	G	G	G	V
201	A	A	A	A
246	I	E	R	V

Figure S1. Alignment of cathepsin B peptidases. A. Alignment of the primary sequences of cathepsin B peptidases from *Fasciola hepatica*, FhCB1 (Q8I7B2) FhCB2 (A5X493) and FhCB3 (A7UNB2) with the major cathepsin B1 peptidase of *Schistosoma mansoni,* SmCB1 (Q8MNY2). The bar shows the position of the occluding loop. Based on SmCB1 mature enzyme numbering the positions associated with the two His residues (His 110 and His 111) and the S2 active site residues, are highlighted in yellow and green, respectively. **B.** Table highlighting the residues that form the S2 active site, in FhCB1, FhCB2, FhCB3 and FhCL1 highlighted in green in the above alignment (based on the SmCB1 mature enzyme numbering, Jílkova *et al.,* 2011).
