# Supplementary figures and images for: The Zoonotic Helminth Parasite Fasciola hepatica: Virulence-Associated Cathepsin B and Cathepsin L Cysteine Peptidases Secreted by Infective Newly Excysted Juveniles (NEJ)
Source: Animals (Basel). 2021 Dec 8;11(12):3495. doi: 10.3390/ani11123495 (PMC8698070; doi:10.3390/ani11123495)

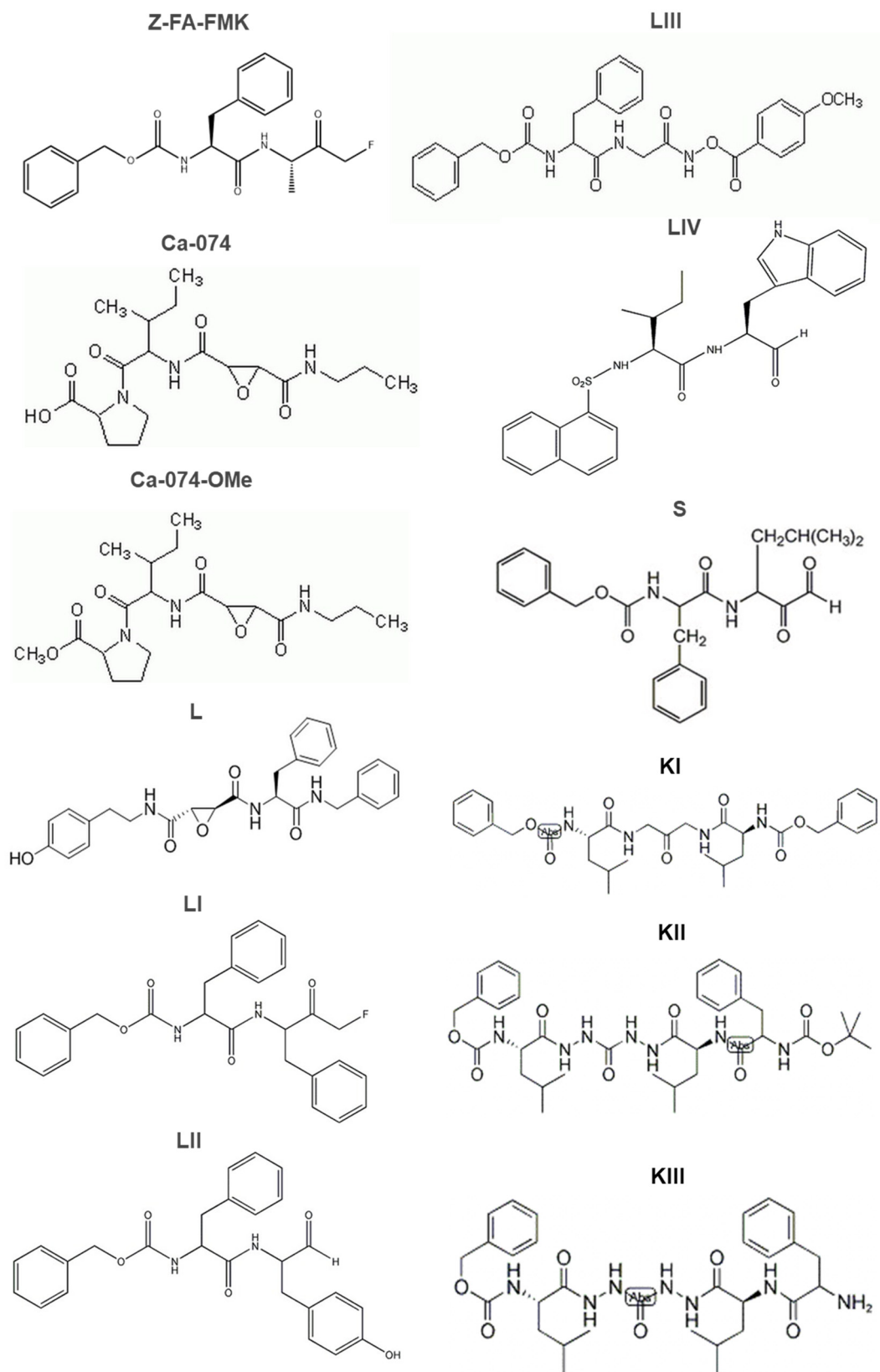

**Figure S2. Structure of the commercial cathepsin inhibitors.**

Supplement: Supplementary file 1 [file animals-11-03495-s001.zip › Supplementary Fig 2.pdf]
